# Comparison and evaluation of experimental mediastinitis models: precolonized foreign body implants and bacterial suspension inoculation seems promising

**DOI:** 10.1186/1471-2334-6-76

**Published:** 2006-04-25

**Authors:** Gulden Ersoz, Barlas Naim Aytacoglu, Nehir Sucu, Lulufer Tamer, Ismet Bayindir, Necmi Kose, Ali Kaya, Murat Dikmengil

**Affiliations:** 1 Department of Clinical Microbiology and Infectious Diseases, Mersin University, School of Medicine, Mersin, Turkey; 2 Department of Cardiovascular Surgery, Mersin University, School of Medicine, Mersin, Turkey; 3 Department of Biochemistry, Mersin University, School of Medicine, Mersin, Turkey

## Abstract

**Background:**

Post-sternotomy mediastinitis (PSM) is a devastating surgical complication affecting 1–3% of patients that undergo cardiac surgery. *Staphylococcus aureus *is one of the most commonly encountered bacterial pathogen cultured from mediastinal samples obtained from patients with PSM. A component of the membrane of the gram positive bacteria, lipoteichoic acid, stimulates the blood monocytes and macrophages to secrete cytokines, radicals and nitrogen species leading to oxido-inflammatory damage. This seems to be responsible for the high mortality rate in PSM. For the evaluation of the pathogenesis of infection or for the investigation of alternative treatment models in infection, no standard model of mediastinitis seems to be available. In this study, we evaluated four mediastinitis models in rats.

**Methods:**

The rats were divided into four groups to form different infection models. Group A: A suspension of 1 × 10^7 ^colony-forming units *Staphylococcus aureus *in 0,5 mL was inoculated from the right second intercostal space into the mediastinum. Group B: A hole was created in the right second intercostal space and a piece of stainless-steel implant with a length of 0.5 cm was inserted into the mediastinum and a suspension of 1 × 10^7 ^cfu bacteria in 0,5 mL was administered via the tail vein. Group C: Precolonized stainless-steel implant was inserted into the mediastinum. Group D: Precolonized stainless-steel implant was inserted into the mediastinum and the bacteria suspension was also injected into the mediastinum. On the 10^th ^day, rats were sacrificed and the extension of infection in the mediastenae was evaluated by quantitative cultures. Myeloperoxidase activity (MPO) and malondialdehyde (MDA) levels were determined in the sera to evaluate the neutrophil activation and assess the inflammatory oxidation.

**Results:**

The degree of infection in group C and D were 83.3% and 100% respectively (*P *< 0.001). MDA levels were significantly higher in these two groups than the others (*P *< 0.001).

**Conclusion:**

Infected implants and high bacterial concentration administration were the two important components that played a significant role in the outcome of a successful infection in mediastinum in a rat model.

## Background

Post-sternotomy mediastinitis (PSM) occurs in 1–3% of all patients undergoing median sternotomy [1]. This devastating complication carries a high risk of morbidity, prolonged hospitalization, increased costs, as well as high mortality that can reach to as high as to 40% [2,3]. Staphylococci are the most common pathogens, accounting for almost 54–82% of the cases of PSM [2,4,5]. *Staphylococcus aureus *and coagulase negative staphylococci are the most frequently encountered bacteriae causing serious infections in connection with the implanted protheses and foreign bodies [6,7]. *S. aureus *has emerged as the major pathogen with more virulence than the other staphylococcal strains. The clinical presentation of mediastinitis caused by the *S. aureus *has been observed to be a severely destructive infection for the immune system with strong signs of systemic inflammation in many of the patients. *S. aureus *may form a biofilm on an avascular and metallic surface, as in implants, giving rise to infections at surgical sites that are difficult to eradicate from the surrounding tissues and bone [8]. When the clinical applications are concerned, the removal of the foreign bodies frequently becomes obligatory in such instances. Definitive diagnosis and treatment of mediastinitis requires reoperations of the mediastinum for direct inspection and thorough debridement. Considering the difficulty in eradication of these infections, experimental animal models have been proposed involving either the inoculation of a bacterial suspension at the anticipated infection site in order to produce a biofilm, or the use of pre-colonized metals.

In order to investigate the role of the immune system in mediastinitis and to propose new medical and surgical approaches in the treatment, we need to have new animal models of mediastinitis. The aim of this study was to investigate whether a mediastinal infection could successfully be created in rats by using *S. aureus*.

## Methods

### Microorganism and precolonization

In this study a methicillin-resistant *Staphylococcus aureus *(MRSA) strain, which has been the responsible agent for a PSM after an open-heart surgery, was used to induce mediastinitis in rats and *S. aureus *ATCC 25923 was used as a control for methicillin susceptibility test.

Stainless steel (DG 316 L monofilament stainless steel non-absorbable suture, No: 5, B&S Tyco/Healthcare/United States Surgical, United Kingdom) were cut into pieces of 0.5 cm long. All of the steel wire pieces were autoclave sterilized before precolonization. They were then placed in tubes containing 2 mL of soy broth to which 2% glucose was added. Fifty microliters of a stationary culture of MRSA (2 × 10^9^cfu/mL) was also added to each tube [9]. Precolonization of the steel wire pieces was allowed to develop for 12 h at 37°C. The steel wire pieces were removed with tweezers under sterile conditions. Following three steps of washing with saline solution to remove unbound bacteria, the pieces were stored at 4°C under sterile conditions during the transfer to the experiment laboratory and until the time of surgery.

To control the presence of precolonization, six pre-colonized pieces were placed in a tube containing 2 mL of saline and vortexed, and 100 μL of solutions were plated onto 5% blood agar. The mean of the quantitative culture results was 608.33 ± 131.97 cfu/mL (450.00 – 800.00 cfu/mL).

The bacteria suspension to be inoculated at the time of surgery, was obtained after an 18 h culture in 5% blood agar plate, washed in saline solution for three times and resuspended to 2 × 10^7^cfu/mL. Each animal received 0.5 mL of this suspension, i.e. 10^7^cfu/mL.

### Animals

This study received the approval of the Ethics Committee of our institution. All animals received humane care according to the European Convention on Animal Care. We used 24 male Sprague-Dawley rats weighing between 190 and 240 g. The rats were kept in standard laboratory cages for 1–2 weeks with free access to standard laboratory diet and water before the beginning of the experiment. During the experiment period they were maintained on a 12-hour light-dark cycle, on 45–55% relative humidity and at 21°C.

### Experimental models

The rats were randomly divided into four groups; n = 6 in each group. The groups were defined as follows:

Group A: Bacteria suspension was instilled from the right second intercostal spaces into the mediastinal cavities.

Group B: A hole was created in the right second intercostal space. A piece of stainless-steel implant, which was 0.5 cm long, was inserted into the mediastinal cavities. The bacteria suspension was given via the tail veins.

**Figure 1:**
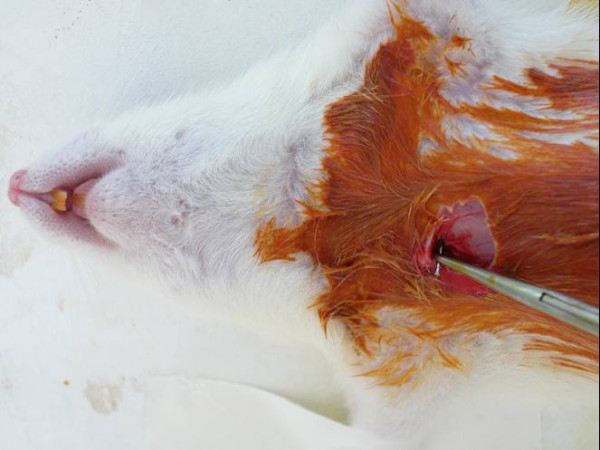
Insertion of the precolonized stainless-steel implants into the mediastinal cavities.

Group C: Precolonized stainless-steel implants were inserted into the mediastinal cavities (Figure 1).

Group D: Precolonized stainless-steel implants were inserted into the mediastinal cavites and bacteria suspension was also instilled into the mediastinal cavities as well.

### Surgical procedure

The rats were anesthetized by an intramuscular injection of 50 mg/kg ketamine hydrochloride. Following skin disinfection of the surgical zone with 10% polyvinylpyrrolidone-iodine, a hole was created in the right second intercostal spaces without access to pleural spaces and the pre-colonized stainless-steel implants were inserted into the right parasternal areas. In group D subjects, the bacteria solution was also inoculated into the mediastinal spaces as well. The incisions were closed with 4/0 silk sutures (Silkam^®^, Braun; Tuttlingen, Germany).

### Assessment of the infection status of the animals

Ten days after the bacterial inoculation, the rats were sacrificed by the injection of high dose intraperitoneal pentothal. A median sternotomy was performed for each rat and 5 mL of blood was aspirated from the hearts and was divided into two tubes (2 mL each). One of them was incubated under aerobic conditions for 48 hr at 37°C in 5 mL of tryptic soy broth and the other tube was sent for biochemical analysis. Pericardium, thymus, lung and mediastinal vessels were harvested, and all tissues were divided into small pieces, weighed separately and placed in a sterile grinding tube. The samples were homogenized with 1 mL of tryptic soy broth using sterile ground-glass stoppers. After mechanical grinding (Pottere S, Biolab, Melcungen-Germany), 500 μL of homogenate was transferred into a tube containing 4.5 mL of 0.9% NaCl, and was used to perform four serial dilutions, the final dilution being 10^-4^. From this dilution, 100 μL aliquots were plated onto two different plates, including tryptic soy agar with 5% blood and EMB agar. Quantitative culture results were determined by the number of colony-forming units per gram calculated from the dilutions of tissue homogenate cultures using the formula [(number of cfu × reciprocal of dilution × 10)/weight of tissue]. To prevent contamination of the environment, these cultures were prepared as the last procedure. All agar plates were incubated under aerobic conditions in 5% CO_2 _for 24 hr at 37°C. Staphylococci were identified by catalase and the tube coagulase tests and methicillin susceptibility test was performed. All strains were identified to be MRSA.

### Biochemical evaluation

#### Determination of MDA

Blood samples were collected into tubes that contained EDTA. Following centrifugation at 3000 rpm for 10 min at +4°C, the obtained plasma was stored at -20°C until analyses. Plasma MDA levels were determined by using reagent kit for HPLC analysis of MDA (Chromosystems, GmbH Germany). Analyses were performed with isocratic HPLC system with fluorescence detection (HP 1100). All solvents were HPLC grade. MDA levels were interpreted as μmol/L.

#### HPLC condition for MDA

Injection volume: 20 μL Flow rate: 1.0 mL/min, Run time: < 5 min, Column and room temperature: 25°C, fluorescence dedector: EX 515 nm, EM 553 nm [10,11].

#### Measurement of MPO

Blood samples were collected into tubes that contained EDTA. Following centrifugation at 3000 rpm for 10 min at +4°C, obtained plasma was stored at -20°C until analyses. Plasma MPO activity was assessed by measuring the H_2_O_2 _dependent oxidation of O-dianosidine. Plasma samples were incubated with 0.2 mg/mL O-dianosidine and 158 μM hydrogen peroxide in 50 nM potassium phosphate buffer (pH = 7.0) at a ratio of 4:1, and changes in absorbance were detected at 410 nm using a microtiter photometric plate reader (Microwell System Reader 230 S; Organon Technica Corp., Durham, N. C.) Enzyme activity was interpreted as U/mL [12].

### Statistical analysis

Data analyses were performed by using the Statistical Package Program SPSS 9.05. The differences in the incidence of mediastinitis among the groups were assessed by χ^2 ^test. Statistical significance was considered to be achieved at a probability of ≤ 0.05. Quantitative cultures, MPO activity and MDA levels were compared by Kruskall-Wallis variance analysis. After Kruskal Wallis analysis, Mann Whitney U test was used to assess the magnitude of infection and the differences in the number of organs involved between the groups. For multiple comparisons, statistical significance was set as K/4 (0.0125) in order to maintain an overall significance level of 0.05.

## Results

**Figure 2:**
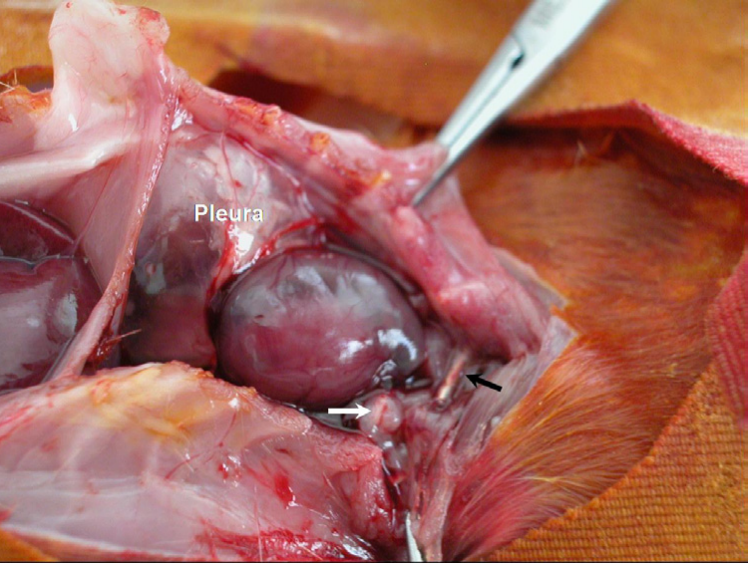
Fibrin bands in the mediastinal cavities of the infected rats (white arrow), prominent pus between the pleural layers and foreign body (stainless steel wire) is seen (black arrow).

As a subjective macroscopic finding we observed tenderized mediastinal tissues. Fibrin adhesions were also present in the mediastinal cavities of the rats that were highly infected (Figure 2). On the other hand, it was macroscopically not quite easy to distinguish between the infected and non infected rats. The number of organs containing viable bacteria and the incidence of the mediastinitis detected in the groups is shown in Table 1. The incidence of mediastinitis was higher in Groups C and D (5/6 in group C; 6/6 in group D) than the other groups (1/6 both in groups A and B) and the difference was reaching to a statistical significance (*P *< 0.05). Bacteremia in the blood cultures were mostly detected in group D, but this was not statistically significant (*P *= 0.204). Bacterial concentrations on tissues, presented as logarithm of geometric mean and standard deviation (Table 2), were not significantly different between the groups (mean, *P *= 0.117; total, *P *= 0.109).

**Table 1:** Number and infection rates of the mediastinal tissues and all infected rats

Groups		Samples						All infected rats^a^	
				
		Tissue		Blood		Steel			
		n	%	n	%	n	%	n	%
Group A	Grown	1	16.7	1	16.7	-	-	1	16.7*
n = 6	Not grown	5	83.3	5	83.3	-	-	5	88.3

Group B	Grown	1	16.7	1	16.7	1	16.7*	1	16.7*
n = 6	Not grown	5	83.3	5	83.3	5	83.3	5	83.3

Group C	Grown	3	50	0	0	2	33.3*	5	83.3*
n = 6	Not grown	3	50	6	100	4	66.7	1	16.7

Group D	Grown	4	66.7	3	50	6	100*	6	100*
n = 6	Not grown	2	33.3	3	50	0	0	0	0

^a ^Bacterial growth from at least one of the maediastinal organs, blood or steel wire has been taken as presence of infection.

* Differences between groups: rate of growth from the steel wire pieces between B and D; *P *= 0.005, rate of growth from steel wire pieces between C and D; *P *= 0.019, rate of total growth between A and C; *P *= 0.027, rate of total growth between A and D; *P *= 0.005, rate of total growth between B and C; *P *= 0.027, rate of total growth between B and D; *P *= 0.005.

**Table 2:** Quantitative bacteria levels, grown from the mediastinal tissues; total and mean (log 10 cfu/g). There were no significant differences between the groups (*P *> 0.05)

**Groups**	**Rat numbers with grown**	**Bacterial concentration (log 10 cfu/g)**				Total*	Mean ± SD**
				
		**Thymus**	**Lung**	**Mediastinal vessels**	**Pericardium**		
A	1	4.78	5.82	-	6.19	6.36	5.88 ± 5.87

B	1	4.65	4.52	3.44	-	4.91	4.43 ± 4.34

C	1	-	-	3.91	-	3.91	3.91
	2	4.81	4.05	5.33	4.9	5.57	4.96 ± 4.93
	3	5.11	-	5.11	-	5.41	5.11

D	1	5.08	5.52	5.65	-	5.95	5.48 ± 5.22
	2	-	6.19	-	5.89	6.36	6.06 ± 5.74
	3	-	-	-	5.76	5.76	5.76
	4	5.48	4.89	6.32	-	6.4	5.92 ± 6.05

* Total: total of log 10 cfu/g of all tissues for each rat ** Mean: mean of log 10 cfu/g of each mediastinal tissue

**Figure 3:**
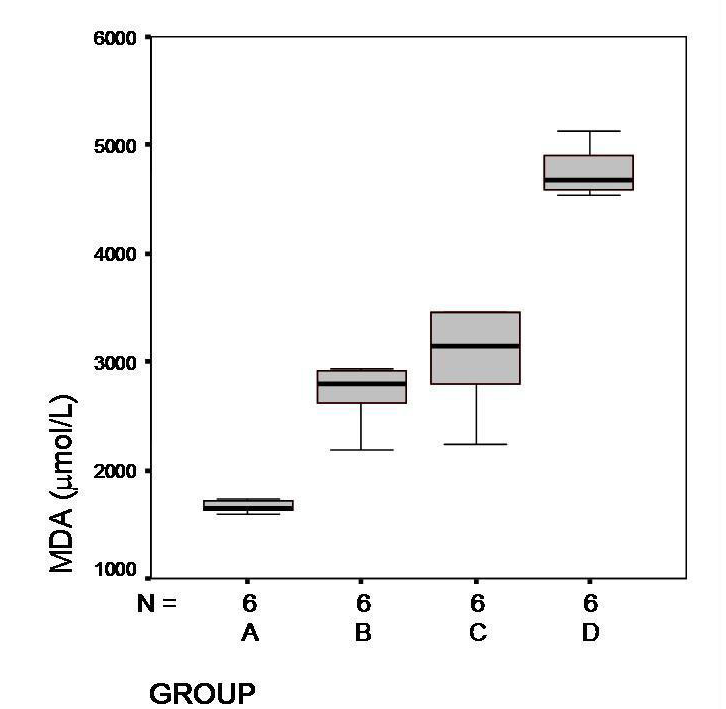
Serum MDA levels and mean values in each groups (μmol/L). Group D, significantly higher than the other groups (*P *< 0,001)

**Figure 4:**
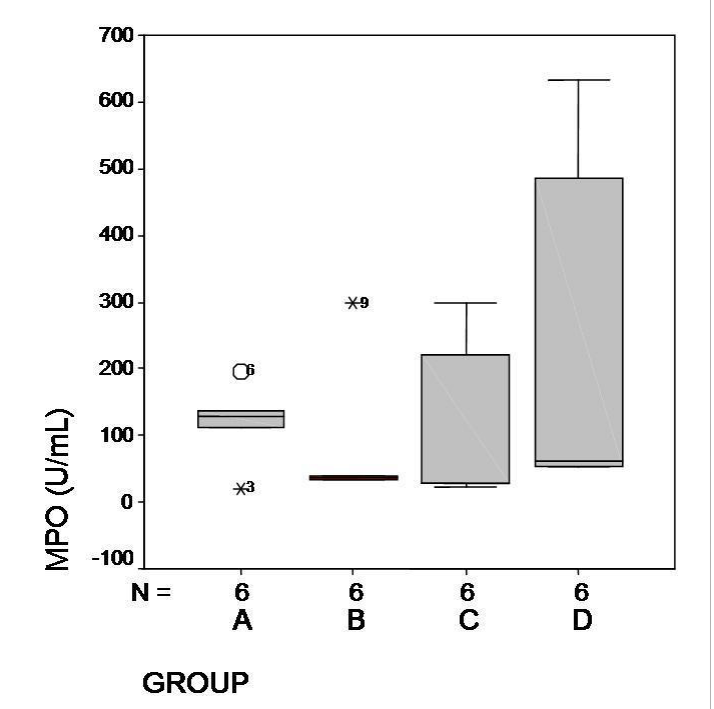
Serum MPO levels and mean values (U/mL). Levels in Group C and D were higher but no statistical significance was found between the groups (*P *= 0, 42).

MDA and MPO levels are shown in Figure 3 and 4 respectively. In Group D, MDA levels were significantly higher than the other groups (*P *< 0,001). The MPO levels were higher in Group D but there were no statistical significance between the other groups (*P *= 0,42).

## Discussion

PSM management generally consists of re-operations with thorough debridement and drainage, followed by a prolonged antibiotic therapy and hospitalization. *S. aureus *is the mostly encountered responsible agent. Carrier et al reported an incidence of 64% MRSA which was responsible for PSM in a cardiovascular intensive care unit where there was no known endemic with MRSA, and isolated MRSA in 1/3 of the patients [13]. In Mekontso-Dessap et al study, they compared the outcome of PSM caused by methicillin-resistant and methicillin-susceptible *S. aureus *[14]. PSM-related death and treatment failure were significantly higher in the MRSA group than in the MSSA group and they found MRSA was the only independent risk factor for overall mortality. The clinical outcome of PSM caused by MRSA is poorer than that caused by MSSA. Peacock et al studies of experimental infection in mice have shown MRSA and MSSA strains to be equally virulent [15]. MRSA and MSSA strains have been shown to possess similar virulence factors, adherence properties, and bacteriological pathogenicity. Duckworth and Jordens found a significantly less attachment of MRSA to cells and fibronectin compared with methicillin-sensitive strains [16]. *S. aureus *strains display a variety of surface protein adhesions collectively known as microbial surface components recognizing adhesive matrix molecules. Fibronectin-binding protein plays prominent roles in *S. aureus *attachment and colonization of host tissues or implanted biomaterials [17]. Therefore, differences in microbial virulence remain controversial, but they might have contributed to the results observed in Mekontso-Dessap et al study [14]. This difference might be explained by several factors, including patient co-morbidities, intrinsic microbial virulence of MRSA strains, and difficulties in achieving effective antimicrobial therapy.

There are many topics to be investigated on the regulation and expression of some major virulence factors, which play an important role in bacterial adhesion, colonization, infection and new bactericidal treatment models. This is why we need to define a standard experimental model for post-sternotomy mediastinal infections with MRSA.

In this study, the 3^rd ^and 4^th ^models, namely Groups C and D, were established successfully with approximately 83% and 100% success rate respectively, as demonstrated bacteriologically.

Our first model using direct inoculation into the mediastinae did not end up with infection over a predetermined time course. Previously, experts for animal experimentation reported that the rats are considerably less susceptible to many of the human pathogens [18]. In a pilot model by Lucet et al, an experimental foreign body infection and therapy was reported. This pilot study was performed to evaluate the subcutaneous injection of 10^8 ^cfu of bacteria that could not produce any infection. However, the study demonstrated that 10^5 ^cfu bacteria could give rise to an infection leading to production of a persistent infection of over ninety five percent of the animals [19].

The second model was simulated by using pieces of non-absorbable steel wire suture as local foreign bodies and bacteria coming from the cardiac pump or other equipment, that were related with circulation. We thought that this model may be simulating the hematogen spreading of infections but this did not turn out to be a successful way for producing mediastinitis.

The last two models were simulated by the administration of bacteriae locally together with foreign bodies. The major differences in the results of the two models were, in group D; the bacterial load was higher than the other. Some conditions such as the duration of the surgery, cardiac resuscitation or re-exploration with re-sternotomy lead to a higher bacterial contamination in the mediastinum. Nishida et al reported that, high infection rate is the result of the poor blood supply of the presternal soft tissues and subjection to excessive tissue damage during the procedure [20]. They performed a canine post-operative mediastinitis model which was a highly traumatic procedure by using the electrocautery on the sternotomy incision. The authors also showed that mediastinitis is encountered significantly more in subjects where circulation is negatively affected and where tissue degeneration is higher [20].

Mediastinitis has encouraged the researchers to develop animal mediastinitis models that would characteristically simulate complex pathologic and pathophysiologic mechanisms of PSM, provide evaluation of pharmacokinetic and antimicrobial effects of the antibiotics (especially, since the worldwide emergence of multiresistant bacteria after postcardiac surgery), and establish new adjuvant treatment strategies (e.g., use of antiinflammatory and antioxidant agents). Our study has several advantages over the previous mediastinitis models, including the greater convenience of rats for extensive antibiotic trials.

Our primary aim was to develop a mediastinitis model. We did not perform any procedure to evaluate the biofilm produced on the surface of the stainless steel by the *S. aureus*. We only measured whether the stainless steel pieces were pre-colonized or not. The previous studies with precolonization and measurement of the biofilm on the surfaces of the foreign bodies lead us to pre-colonize the stainless steel pieces [8,21]. We checked for the precolonization and all stainless steels were precolonized successfully.

By the stimulation of blood neutrophils, monocytes and macrophages by a component of the membrane of the gram positive bacteria, such as lipoteichoic acid (LTA) of *S. aureus*, cytokines, free oxygen radicals and nitrogen species are secreted [22]. This leads to an oxido-inflammatory damage, which appears to be responsible for the high mortality rate [22,23]. Non-enzymatic lipid peroxidation is an example of a free radical associated process through which oxidative stress promotes cellular damage [24]. MDA is the breakdown product of the major chain reactions leading to significant oxidation of such polyunsaturated fatty acids as linolenic acids and thus serves as a reliable marker of oxidative stress [25]. In this study, elevated levels of MPO and consequently MDA were observed. Activity of neutrophils and endothelial cells were detected by increased levels of MPO. Elevated levels of MDA levels were also detected in group D. All these data suggest that even a remote mediastinitis may result with increased oxidative status in systemic circulation. Development of vascular injury was correlated with the tissue accumulation of neutrophils. The high levels of MPO activity clearly demonstrated that the activation of neutrophils and the MDA levels increased after mediastinitis. The major limitation of this study was the absence of establishing the MPO and MDA levels in the mediastinal organs. This is mainly due to physical dimensions of the rat organs, which are quite small. However, this study was a pilot study where MPO and MDA levels were evaluated in the systemic circulation which turned out to be a trustworthy guide for our future studies in the evaluation of mediastinitis and treatment models.

To our knowledge, this study is the first to report on an experimental animal model for mediastinitis.

## Competing interests

The author(s) declare that they have no competing interests.

## Authors' contributions

GE carried experimental and microbiologic studies, participated in the sequence alignment and drafted the manuscript, in the design of the study and performed the statistical analysis. BNA carried experimental process and participated in the sequence alignment and drafted the manuscript. NS participated in the design of the study and in the sequence alignment. LT carried biochemical studies. IB carried experimental and microbiologic studies. NK carried experimental process. AK and MD participated in the design of the study conceived of the study, and participated in its design and coordination. All authors read and approved the final manuscript.

We uploaded the figures as separate figure files form on the submission system, and deleted the figure from our manuscript file.

## Pre-publication history

The pre-publication history for this paper can be accessed here:


